# Psychometric Evaluation of the FFOCI–SF and Other Clinical Outcome Measures in a Group Therapy for Overcontrol (Group Radical Openness)

**DOI:** 10.1002/mpr.70069

**Published:** 2026-03-07

**Authors:** Conal Twomey, Amelia Nelson‐Sisinni

**Affiliations:** ^1^ Psychology Department St Patrick's Mental Health Services Dublin Ireland; ^2^ School of Psychology University College Dublin Dublin Ireland

**Keywords:** convergent validity, exploratory factor analysis, FFOCI‐SF, group psychotherapy, overcontrol, reliability

## Abstract

**Objectives:**

Group Radical Openness (GRO) is a group therapy program targeting costly and harmful overcontrol. This service review psychometrically evaluated GRO's outcome measures as part of the clinical team's ongoing deliberation about their suitability. Particular attention was given to the Five Factor Obsessive–Compulsive Inventory–Short Form (FFOCI–SF), the program's primary measure of overcontrol.

**Method:**

Routine pre‐intervention clinical outcome data from 241 GRO participants were analysed. Internal consistency was examined for all outcome measures. Associations between the FFOCI‐SF and theoretically‐related secondary measures were calculated to assess convergent validity. An exploratory factor analysis (EFA) was conducted on the 48 FFOCI‐SF items. Associations of emergent factors with secondary measures were also computed.

**Results:**

The FFOCI‐SF showed excellent internal consistency (*α* = 0.92) and small‐to‐moderate associations with secondary measures of rigidity, relationship distance, and emotional suppression, supporting convergent validity. Reliability of secondary measures was more variable, with some subscales below accepted thresholds. EFA of the FFOCI‐SF revealed a four‐factor solution—Excessively High Standards, Need for Structure, Overthinking, and Disconnection from Self/Others—each factor yielding good‐to‐excellent internal consistency and theoretically consistent associations with the secondary measures.

**Conclusion:**

These findings support the current outcome‐measurement framework used in GRO while highlighting areas where refinement of secondary measures may be warranted. They also extend understanding of overcontrol by identifying a four‐factor structure within the FFOCI‐SF that aligns with GRO's therapeutic model and provides a foundation for future measure development and evaluation.

## 1 Introduction

Within a well‐supported theoretical framework of personality, individuals characterised by overcontrol—in contrast to undercontrol—tend to be restrained, inhibited, highly organised, socially avoidant, emotionally suppressive, and prone to delaying gratification (Alessandri et al. 2014; Block and Block 1980; Bohane et al. 2017; Robins et al. 1996). General population research has shown that overcontrol is relatively common; yet clinical research around prevalence and intervention is relatively limited, perhaps because the closely related constructs of ‘high self‐control’ and ‘extreme conscientiousness’ seemingly have the potential to be adaptive in certain domains such as organisation and academic achievement (Carter et al. 2016; Clemente et al. 2022; Tangney et al. 2004; Volkert et al. 2018). It is clear, however, that overcontrol is often costly and harmful: Associations have been demonstrated for social isolation, relational distance, cognitive rigidity, and hyper‐perfectionism, and overcontrol underpins a range of clinical presentations including mood and eating disorders, obsessive‐compulsive personality disorder, and avoidant personality disorder (Bohane et al. 2017; Booth and Egan 2023; Gilmartin et al. 2024; Isaksson et al. 2021; Lynch et al. 2015; Lynch et al. 2020).

Group Radical Openness (GRO) is a recently developed 27‐session group therapy program for costly and harmful overcontrol, with initial studies supporting its feasibility and acceptability (Booth and Egan 2023; Egan et al. 2021; Johnstone et al. 2024). A recent service evaluation (*n* = 101) also supported the overall effectiveness of GRO for reducing symptoms of overcontrol and general psychological distress (Twomey et al. 2025).

In contrast to the skills‐based approach of Radically Open Dialectical Behaviour Therapy (Lynch et al. 2020), GRO emphasises interpersonal processes and co‐regulation, drawing from Polyvagal Theory (Dana 2018; Porges 2011), and positions close and authentic relationships among group members as the primary driver of change (Egan et al. 2021). GRO also aims to increase awareness of overcontrol and its impacts, psychological flexibility, and emotional openness (Booth and Egan 2023). Structurally, the first 15 sessions emphasise group exercises and reflective work on three core themes of overcontrol—distance in relationships, rigidity, and inhibited emotion—while the final 12 sessions shift toward experiential and behavioural change.

As with other interventions, GRO undergoes continuous review at both clinical team and organisational levels for quality assurance, within its host setting—St Patrick's Mental Health Services (SPMHS), an independent, not‐for‐profit mental health service in Ireland. An important element of this review is monitoring the suitability of clinical outcome measures, particularly given that GRO is a relatively new intervention with outcome targets that may evolve over time.

The selection of clinical outcome measures in GRO was guided by a review of existing overcontrol measures, and by their alignment with the three themes of overcontrol addressed in the program (Booth and Egan 2023). The team decided upon the Five‐Factor Obsessive‐Compulsive Inventory—Short Form (FFOCI‐SF) (Griffin et al. 2018) as the primary measure of overcontrol. The Revised Adult Attachment Scale—Close Relationships Version (RAAS) (Collins 1996) addressed the theme of relational distance; the Personal Need for Structure Scale (PNS) (Neuberg and Newsom 1993) addressed rigidity; and the Emotion Regulation Questionnaire (ERQ) (Gross and John 2003) addressed inhibited emotion. A general measure of psychological distress was also included: the Brief Symptom Inventory (BSI) (Derogatis and Melisaratos 1983).

A previous service evaluation (which involved a subset of GRO participants as it encompassed a comparative analysis of in‐person and online versions) yielded generally good internal consistency for GRO's outcome measures, though concerns were raised around some of the secondary measures (Twomey et al. 2025). There is also ongoing deliberation among the clinical team about outcome measure suitability and the potential need for measure refinement or replacement. In this context, the present service review psychometrically evaluated GRO's outcome measures. Particular attention was given to FFOCI–SF as the program's primary measure of overcontrol.

## 2 Method

### 2.1 Participants

This service review, entailing a psychometric evaluation of GRO's outcome measures, availed of routinely collected (anonymised) clinical data from an independent, not‐for‐profit mental health service in Ireland: SPMHS. It was reviewed and approved by SPMHS. Participants were SPMHS service users who attended the GRO program across 28 group cycles (15 delivered in‐person; 13 via videoconferencing) between January 2020 and July 2025.

Regarding the screening process, psychologists within the SPMHS network of multidisciplinary teams completed a GRO pre‐screening form and referred patients who exhibited strong characteristics of overcontrol. These individuals then underwent a comprehensive semi‐structured interview conducted by two members of the GRO team (a clinical or counselling psychologist and an assistant psychologist).

### 2.2 Measures

All of GRO's outcome measures are completed by self‐report. GRO's primary measure of overcontrol is the FFOCI‐SF, a 48‐item questionnaire assessing maladaptive overcontrol traits based on the Five‐Factor Model of personality (Griffin et al. 2018). The FFOCI‐SF has 12 subscales and a total score; however, only the total score is used in GRO, as the program developers opted to reduce complexity and assess the subdomains of overcontrol linked to GRO's three core themes using secondary measures (Booth and Egan 2023). Furthermore, use of the total score reflects GRO's non‐diagnostic, continuum‐based focus on overcontrol and its broad scope beyond any single diagnostic definition. The FFOCI‐SF has demonstrated strong internal consistency and convergent validity (e.g., robust correlations with corresponding Five‐Factor Model scales) in general population samples (Grey et al. 2024; Griffin et al. 2018; Kłosowski et al. 2019).

As noted, three secondary measures are applied to the core domains of overcontrol specifically targeted in GRO. Addressing relational distance, the 18‐item RAAS measures adult attachment styles through subscales relating to relational intimacy, comfort with depending on others, and interpersonal anxiety (Collins 1996). Acceptable levels of reliability and validity have been indicated for all three subscales in general population samples (Collins 1996; Teixeira et al. 2019; Troisi et al. 2022). Addressing rigidity, the 11‐item PNS assesses an individual's preference for order and certainty through two related factors: Desire for Structure and Response to a Lack of Structure (Neuberg and Newsom 1993). Sufficient reliability and validity levels for this measure have been demonstrated in general population samples (Gil et al. 2024; Hamtiaux and Houssemand 2012; Neuberg and Newsom 1993; Shi et al. 2009). Addressing inhibited emotion, the 10‐item ERQ has two subscales—cognitive reappraisal and expressive suppression—and it has yielded good support for its internal consistency and validity in non‐clinical samples (Gross and John 2003; Preece et al. 2020).

Regarding GRO's general distress questionnaire, the BSI is a well‐established measure of general psychological distress symptoms across 53 items, and its psychometric properties have been supported in numerous general and clinical populations (Adawi et al. 2019; Derogatis and Melisaratos 1983; Endermann 2005). The BSI's Global Severity Index is used for GRO outcome measurement purposes.

### 2.3 Statistical Analysis

Statistical analyses of pre‐intervention data were performed using SPSS version 29. Internal consistency was assessed for all outcome measures. Simultaneously addressing convergent validity and theoretical coherence, Pearson's *r* correlations were calculated between the FFOCI‐SF and the secondary measures reflecting GRO's three core themes of overcontrol. An exploratory factor analysis (EFA) was also conducted on the FFOCI‐SF. Associations of emerging factors with secondary measures were subsequently calculated. The analyses were conducted using complete cases (listwise deletion) which can provide unbiased estimates in analyses of reliability and factor structure when missingness is minimal (Newman 2014; Schafer and Graham 2002).

### 2.4 Sample Size

With an expectation of a sample size of 230–250 based on an initial review of available clinical data, this service review was expected to have sufficient power for estimating internal consistency (Bonett 2002), and for detecting medium‐sized intercorrelations (Bonett and Wright 2000). Given the expectation of a relatively small variables‐to‐factors ratio for the EFA, findings from a simulation study indicated that a sample of roughly 180 participants would be necessary (Mundfrom et al. 2005).

## 3 Results

### 3.1 Demographics

A total of 241 GRO participants, aged between 18 and 68 years (*M* = 41.6 years, SD = 13.16) contributed routinely collected clinical outcome data for this service review. There was a well‐balanced gender ratio: 124 females (51.5%) and 117 males (48.5%). 132 participants completed GRO in person, and 109 completed GRO through videoconferencing. Group sizes ranged from 6 to 11 participants. Table 1 displays the mean scores for each outcome measure. The mean FFOCI‐SF score was 165 (out of 240), indicating endorsement of overcontrol traits above the scale midpoint; this may suggest a moderate level, although interpretation is tentative given the absence of published normative references.

**TABLE 1:** Reliability (cronbach's alpha; *α*) of outcome measures for GRO (pre‐intervention).

Measure	*N*	Missing values	Mean (maximum possible)	SD	(*α*)
Primary measure of overcontrol					
FFOCI‐SF	206	35	165.18 (240)	25.56	0.92
Secondary measures of overcontrol					
ERQ: Cognitive reappraisal	235	6	22.36 (42)	7.84	0.82
ERQ: Expressive suppression	236	5	20.31 (28)	4.49	0.60
PNS: Desire for structure	240	1	17.57 (24)	3.98	0.68
PNS: Response to a lack of structure	235	6	32.77 (42)	6.36	0.81
RAAS: Anxiety	238	3	14.50 (30)	4.99	0.83
RAAS: Close	234	7	15.01 (30)	3.42	0.74
RAAS: Depend	237	4	21.97 (30)	5.58	0.43
General distress measure					
BSI: Global severity index (GSI)	202	39	132.38 (265)	37.21	0.96

Abbreviations: BSI = Brief Symptom Inventory, ERQ = Emotional Regulation Questionnaire, FFOCI‐SF = Five Factor Obsessive Compulsive Inventory – Short Form, PNS = Personal Need for Structure, RAAS = Revised Adult Attachment Scale.

### 3.2 Internal Consistency

As shown in Table 1, the FFOCI‐SF demonstrated excellent internal consistency (*α* = 0.92). For GRO's distance in relationships theme, two of the three RAAS subscales showed acceptable or higher internal consistency, whereas the Depend scale fell far below the acceptable threshold (*α* = 0.43). For the rigidity theme, the PNS Response to a Lack of Structure subscale showed good internal consistency (*α* = 0.81), while the Desire for Structure subscale was slightly below the acceptable threshold (*α* = 0.68). For the emotional suppression theme, the ERQ Cognitive Reappraisal subscale demonstrated good internal consistency (*α* = 0.82), whereas the Expressive Suppression subscale was clearly below the acceptable level (*α* = 0.60). Regarding the general distress measure, the BSI Global Severity Index yielded excellent internal consistency (*α* = 0.96). Table S1 presents internal consistency estimates for the FFOCI‐SF subscales, which are not discussed in this paper as they are not used in GRO.

### 3.3 Intercorrelations: FFOCI‐SF Total Score and Secondary Measures

As shown in Table 2, the FFOCI‐SF total score demonstrated significant associations (in the expected directions) with most secondary measure subscales reflecting GRO's core themes of overcontrol. For the distance in relationships theme, small‐to‐moderate FFOCI‐SF associations were yielded for all three RAAS subscales: Close (*r* = −0.36, *p* < 0.001); Anxiety (*r* = 0.26, *p* < 0.001); and Depend (*r* = −0.20, *p* < 0.005). For the rigidity theme, strong associations were yielded for both PNS subscales: Desire for Structure (*r* = 0.55, *p* < 0.001) and Response to Lack of Structure (*r* = 0.58, *p* < 0.001). For the emotional inhibition theme, there was no significant FFOCI‐SF association with ERQ Cognitive Reappraisal (*r* = −0.04, *p* > 0.05), but a small positive association was yielded for ERQ Expressive Suppression (*r* = 0.25, *p* < 0.001). Finally, there was a small‐to‐moderate FFOCI‐SF association with general psychological distress as shown by the BSI Global Severity Index (*r* = 0.29, *p* < 0.001). Table S2 displays associations of the FFOCI‐SF subscales (not used in GRO) with secondary measures.

**TABLE 2:** Associations of FFOCI‐SF total score with secondary measures addressing GRO's core themes of overcontrol, and general distress.

	Theme: Distance in relationships			Theme: Rigidity		Theme: Emotional inhibition		General distress
	RAAS close	RAAS depend	RAAS anxiety	PNS desire for structure	PNS response to lack of structure	ERQ cognitive reappraisal	ERQ expressive suppression	BSI total
FFOCI total	−0.36^a^	−0.20^a^	0.26^a^	0.55^a^	0.58^a^	−0.04	0.25^a^	0.29^a^

Abbreviations: BSI = Brief Symptom Inventory, ERQ = Emotional Regulation Questionnaire, FFOCI‐SF = Five Factor Obsessive‐Compulsive Inventory–Short Form, PNS = Personal Need for Structure, RAAS = Revised Adult Attachment Scale.

^a^ Correlation is significant at the 0.01 level (2‐tailed).

### 3.4 Exploratory Factor Analysis of the FFOCI‐SF

Consistent with the practice in GRO of using the total score, the EFA was conducted on all 48 FFOCI‐SF items. Principal axis factoring with Promax rotation was deployed. Suitability of the FFOCI‐SF data for factor analysis was confirmed in initial tests: the Kaiser–Meyer–Olkin (KMO) measure of sampling adequacy was 0.87, and Bartlett's test of sphericity was significant, *χ* ^2^ (1128) = 5200.97, *p* < 0.001. Initial extraction identified 11 factors with eigenvalues > 1.0, accounting for 55.7% of the variance. However, inspection of both the scree plot and the additional variance explained by each factor suggested a more parsimonious four‐factor solution. The analysis was therefore re‐run with the number of factors fixed at four.

The four‐factor solution is displayed in Table 3. Internal consistency for the four factors was good‐to‐excellent, with Cronbach's *α* ranging from 0.81 to 0.92. This solution accounted for 42.7% of the total variance. Factor 1 was labelled ‘Excessively High Standards’, reflecting excessive focus on work, inflated pride in efficiency and work quality, dogged self‐discipline, hyperdrive for achievement, perfectionism, and over‐responsibility. Factor 2 was labelled ‘Need for Structure’, reflecting reliance on predictability, needing to follow the ‘tried and true’, a strong routine‐orientation, preference for safety and rules, disproportionate risk minimisation, and following rigid moral and ethical codes. Factor 3 was labelled ‘Overthinking’, reflecting proneness to rumination and excess deliberation, tendency to worry about the future and consequences, and excessive focus on minute details. Factor 4 was labelled ‘Disconnection from Self and Others’, reflecting a lack of emotional connection with self and others, limited interpersonal warmth and engagement, and struggles with intimacy.

**TABLE 3:** Exploratory factor analysis of the FFOCI‐SF items (*n* = 206).

F1: Excessively high standards (*α* = 0.92)			F2: Need for structure (*α* = 0.86)			F3: Overthinking (*α* = 0.85)			F4: Disconnection from self/others (*α* = 0.81)		
Item	Content (abbreviated)	Loading	Item	Content (abbreviated)	Loading	Item	Content (abbreviated)	Loading	Item	Content (abbreviated)	Loading
22	Workaholic.	0.86	41	Predictability.	0.83	13	Rumination.	0.81	26	Not a warm person.	0.79
23	Work completion.	0.80	27	Preference for safety.	0.82	48	Deliberation.	0.69	38	Struggle with intimacy.	0.68
47	Doggedness.	0.79	39	Dull life to others.	0.73	25	Worrier.	0.65	2 (r)	Not warm or engaging.	0.63
7	Pride in quality of work.	0.72	17	Tried and true.	0.65	12	Think of consequences.	0.63	4	Not into others' feelings.	0.59
11	Getting things done.	0.71	15	Playing it safe.	0.63	37	Fear of failure.	0.60	28	Emotions unimportant.	0.53
46	Getting ahead.	0.68	9	Play by the rules.	0.57	1	Worry about the future.	0.55	14 (r)	Dislike personal level.	0.48
10	Caught up in my work.	0.64	21	Rules are important.	0.50	24	Examine every detail.	0.52	40	Small range of emotions.	0.48
31	Pride in efficiency.	0.63	29	Preference for routine.	0.49	36	Being sure before I act.	0.49	16	Not emotionally relating.	0.46
44	Work is organised.	0.62	18	Following moral code.	0.49	8	Consider minute details.	0.43			
35	Strong self‐discipline.	0.61	42	Following ethical code.	0.39						
20	Detail‐oriented.	0.59	3 (r)	Dislikes risk.	0.35						
33	Over‐responsibility.	0.58	5	Others find me dull.	0.34						
34	Work is my pleasure.	0.55									
19	Perfectionist.	0.53									
32	Focused on organizing.	0.47									
45	Emphasis on morality.	0.40									
6	Unyielding morals.	0.38									
43	Flawless work.	0.36									
30	Against permissiveness.	0.24									

*Note:* The extraction method was principal axis factoring with Promax rotation. Item wording is adapted from Griffin et al. (2018).

Abbreviation: F = Factor. Reverse‐scored items are denoted with (*r*).

The first three factors—Excessively High Standards, Need for Structure, and Overthinking—were moderately inter‐correlated, with *r* values ranging from 0.38 to 0.47. By contrast, smaller associations of the first three factors with the Disconnection from Self and Others factor were yielded, as shown by the following corresponding *r* values: Excessively High Standards, −0.05, Need for Structure, 0.26; Overthinking, −0.08. This pattern indicates moderate overlap among the first three factors but relative independence of the fourth, supporting the overall four‐factor solution; at the same time, it could be viewed as a ‘three‐plus‐one’ configuration, with the first three factors possibly reflecting a broad ‘rigidity’ dimension that would need to be confirmed in future research.

Table S3 displays the factor solution derived from EFA of the 12 FFOCI‐SF subscales (included as a supplementary file for research purposes beyond the scope of the current GRO service review).

### 3.5 Intercorrelations: FFOCI‐SF Four Factor Model and Secondary Measures

As shown in Table 4, expected associations of the four emerging FFOCI‐SF factors with related dimensions of overcontrol were yielded. Moderate positive associations of the first three factors—which apparently tap into parts of rigidity—with the PNS (GRO's measure of choice for rigidity) were yielded, with *r* values ranging from 0.33 to 0.60. Regarding the fourth factor, Disconnection from Self and Others was associated with both emotional suppression (*r* = 0.29) and the relational closeness subscale from the RAAS (*r* = 0.38). Overthinking (Factor 3) was also significantly associated with all of the RAAS subscales—with *r* values ranging from 0.22 to 0.33—and it yielded the strongest association with general distress as indicated by the BSI Global Severity Index (*r* = 0.48).

**TABLE 4:** Associations of EFA factors with primary measure of overcontrol, secondary measures addressing GRO's core themes of overcontrol, and general distress.

	Theme: Distance in relationships			Theme: Rigidity		Theme: Emotional inhibition		General distress
	RAAS close	RAAS depend	RAAS anxiety	PNS desire for structure	PNS response to lack of structure	ERQ cognitive reappraisal	ERQ expressive suppression	BSI total
Factor 1: Excessively high standards^a^	−0.16^d^	−0.14^d^	0.18^d^	0.47^e^	0.38^e^	0.01	0.16^d^	0.17^d^
Factor 2: Need for structure^a^	−0.23^e^	−0.03	0.16^d^	0.53^e^	0.60^e^	−0.01	0.13	0.14
Factor 3: Overthinking^b^	−0.27^e^	−0.22^e^	0.36^e^	0.33^e^	0.51^e^	−0.04	0.11	0.48^e^
Factor 4: Disconnection from self and others^c^	−0.38^e^	−0.05	0.05	0.11	0.14^d^	−0.08	0.29^e^	0.05

Abbreviations: BSI = Brief Symptom Inventory, ERQ = Emotional Regulation Questionnaire, FFOCI‐SF = Five Factor Obsessive‐Compulsive Inventory – Short Form, PNS = Personal Need for Structure, RAAS = Revised Adult Attachment Scale.

^a^ Hypothesized correlation with Theme 2: Rigidity scales.

^b^ Hypothesized correlations with Theme 1: Distance in Relationships and Theme 2: Rigidity scales.

^c^ Hypothesized correlations with Theme 1: Distance in Relationships and Theme 3: Emotional Inhibition.

^d^ Correlation is significant at the 0.05 level (2‐tailed).

^e^ Correlation is significant at the 0.01 level (2‐tailed).

## 4 Discussion

### 4.1 Summary of Main Findings

This service review provided a psychometric evaluation of GRO's outcome measures as part of the clinical team's ongoing consideration of their suitability and the potential need for refinement or replacement. As GRO's primary indicator of overcontrol, the FFOCI‐SF showed excellent internal consistency. Additionally, significant FFOCI‐SF associations with GRO's secondary measures of overcontrol were yielded, supporting its convergent validity. Findings for the secondary measures were more mixed: only two of the three subscales on the RAAS demonstrated acceptable or higher internal consistency; for both the PNS and the ERQ, only one of the two subscales met this threshold. In contrast, the BSI exhibited excellent internal consistency.

In further analysis, an item‐level EFA of the FFOCI‐SF indicated a four‐factor solution; each factor showed good‐to‐excellent internal consistency and correlated with the secondary overcontrol measures in the expected directions, providing further evidence of convergent validity. The first three factors—Excessively High Standards, Need for Structure, and Overthinking—were moderately intercorrelated whereas small associations with ‘Disconnection from Self and Others’ were yielded. This pattern indicates a potential ‘three‐plus‐one’ configuration, with the first three factors possibly reflecting a broad rigidity dimension that merits confirmation in future research. Among the three rigidity‐related factors, Overthinking yielded the strongest associations with GRO's rigidity measure (PNS), it was also associated with scores on the program's measure of relational distance (the RAAS), and it displayed the strongest general distress association of any of the secondary measures. This pattern tentatively suggests that overthinking may play a particularly important maladaptive role in the rigidity‐related aspects of overcontrol.

### 4.2 Comparison With Other Findings and Theoretical Implications

The strong yielded support for FFOCI‐SF in terms of both reliability and convergent validity aligns with findings from general population samples (Gray et al. 2024; Griffin et al. 2018; Kłosowski et al. 2019) and with other psychometric findings obtained for the longer version of the measure (Crego et al. 2015; Hill et al. 2025; Samuel et al. 2012). The mixed internal consistency observed for GRO's secondary overcontrol‐related measures was not a major surprise, as previous research in general population samples has typically reported reliability estimates that are adequate‐to‐good rather than excellent (Gil et al. 2024; Gross and John 2003; Neuberg and Newsom 1993; Preece et al. 2020; Teixeira et al. 2019). As expected, the already well‐established BSI yielded excellent internal consistency.

Although the present EFA was conducted at the item level, the resulting factor structure showed some correspondence with the original FFOCI‐SF facet composition. In particular, the Disconnection from Self and Others factor comprised items drawn entirely from the Detached Coldness and Constricted facets, while the remaining factors similarly reflected coherent groupings of facet content. Although direct comparability is limited by the use of item‐level rather than subscale‐level analyses, this convergence is consistent with prior subscale‐level findings indicating that Detached Coldness and Constricted content cluster (Kłosowski et al. 2019). Beyond this specific facet‐level convergence, both the present item‐level analysis and these prior subscale‐level findings point toward a broader four‐factor configuration in which three factors reflect a common rigidity‐related domain, while a fourth captures interpersonal–emotional disconnection (Kłosowski et al. 2019). Taken together, these findings point to a tentative ‘three‐plus‐one’ higher‐order structure within the FFOCI‐SF. This possibility merits further investigation using confirmatory approaches. That said, retaining the original 12‐factor solution is also justifiable given previous supportive evidence and a coherent theoretical rationale (Crego et al. 2015; Samuel et al. 2012).

The pattern of findings indicating a particularly important maladaptive role for overthinking in overcontrol may reflect the tendency for persistent rumination, worry and related forms of overthinking to contribute not only to cognitive rigidity but also to interpersonal withdrawal and to elevated general distress—consistent with previously established associations of repetitive rumination with loneliness and depressive mood states (Luo et al. 2025; Nolen‐Hoeksema et al. 2008; Patrichi et al. 2025; Tong et al. 2021).

### 4.3 Clinical Implications

From a service‐review perspective, the findings should bolster confidence in GRO's current outcome measurement framework for three main reasons. First, the FFOCI‐SF total score demonstrated excellent internal consistency and showed the expected convergent associations with secondary overcontrol‐related measures (i.e., RAAS, PNS, and ERQ). Second, viewing these associations from the opposite direction, the RAAS, PNS, and ERQ each represented GRO's three core themes—distance in relationships, relationships, and emotional inhibition—as central to overcontrol, reinforcing the theoretical coherence of the overall measurement framework. Third, the clear psychometric evidence for the BSI Global Severity Index as an indicator of general distress supports its continued use. On the other hand, the more mixed reliability of several secondary measures and particularly weak overcontrol associations for the ERQ, highlights potential areas for refinement or replacement to ensure that all three of GRO's core themes are assessed with similar dependability. Supplementary or alternative measures of relational closeness or emotional expression could, for example, provide a more stable assessment of these domains.

Regarding the EFA findings, the emerging ‘three‐plus‐one’ configuration maps closely onto GRO's existing conceptualisation of overcontrol as comprising rigidity, distance in relationships, and emotional suppression. The clustering of the first three factors offers empirical support for the program's emphasis on rigidity as a central process, while the distinctiveness of the fourth factor reinforces the importance of specifically targeting interpersonal and emotional connection. These findings could invite the clinical team to consider whether minor refinements to the content of the three core themes could enhance the focus of intervention: for example, emphasizing additional common ground between the relational distance and emotional inhibition themes or incorporating a more explicit emphasis on overthinking within the rigidity domain.

Beyond the immediate context of GRO, these findings illustrate how routine clinical data can be used to evaluate and refine outcome measures. The findings supporting the utility of the FFOCI‐SF should provide reassurance for other services considering its use. Moreover, although researchers often focus on its subscales, the FFOCI‐SF total score appears to function well in clinical practice as a global indicator of overcontrol. The emerging ‘three‐plus‐one’ factor structure also contributes to the wider conceptualisation of overcontrol by highlighting the potential for a broad rigidity dimension alongside a distinct interpersonal–emotional disconnection dimension. This may inform future overcontrol intervention development. Finally, the identification of a small number of higher‐order factors may be useful for simplifying outcome measurement in overcontrol interventions, although any move toward alternative scoring or summary composites would require further validation before routine use.

### 4.4 Methodological Considerations

Various methodological issues should be considered when interpreting the findings. First, data were derived from routinely collected, anonymised clinical records; while this reflects real‐world practice and strengthens the study's relevance to service evaluation, it limited the availability of detailed demographic or diagnostic information that might have allowed for more nuanced subgroup analyses. Second, the findings were derived from one clinical service and the EFA used only baseline (cross‐sectional) data, which limits how far the factor structure can be generalised and prevents conclusions around factor stability over time. Third, a confirmatory factor analysis (CFA) was not conducted, as this was beyond the scope of the study's service‐review design. Replication in independent samples is therefore required before firm conclusions about the four‐factor solution can be drawn, particularly with respect to the hypothesised overarching rigidity dimension. Fourth, although conventional indicators supported factorability, the sample size was modest relative to the number of items included in the EFA. While nearly all items comfortably loaded above commonly accepted thresholds (i.e., > 0.30), a small number showed comparatively weaker loadings (e.g., Item 30, relating to permissiveness). Fifth, although internal consistency, convergent validity, and the factor structure of the FFOCI‐SF were examined, other important aspects of psychometric evaluation—such as predictive validity or sensitivity to change—were beyond the scope of this service review and remain to be investigated in the context of GRO and similar therapeutic programmes in clinical settings. That said, recent service evaluations of GRO have demonstrated significant pre–post reductions in FFOCI‐SF total scores, supporting its sensitivity to change in this therapeutic context (Twomey et al. 2025). Finally, although the FFOCI‐SF is commonly interpreted at the facet level, GRO uses the total score pragmatically as a global indicator of overcontrol. While this approach capitalises on the breadth of the measure, it necessarily obscures facet‐level distinctions and should not be interpreted as a recommendation for general use of the total score outside this clinical context.

### 4.5 Future Research

The newly derived subscales from the four‐factor solution offer a promising avenue for updating GRO's secondary outcome measures, but they first require further validation through a CFA in an independent clinical sample. Such validation could be built into routine outcome monitoring in GRO as additional data accumulate and could also be explored in general population or other clinical samples. More broadly, future research may examine whether a reduced set of three or four higher‐order factors could be developed into streamlined, clinically oriented composites. Given the modular structure of the FFOCI‐SF, such composites may offer a pragmatic alternative in contexts where the full facet structure is considered overly complex, while remaining flexible to different conceptualisations of overcontrol. However, any such refinement would require confirmatory testing and evaluation of clinical utility before being considered for implementation.

In parallel, qualitative findings on participants' perceived mechanisms of change—such as Safety and Connection, Understanding Overcontrol, and Carrying GRO Forward (Johnstone et al. 2024)—could be combined with these psychometric results to refine both outcome measurement and the program's therapeutic focus. Integrating participant‐reported mechanisms of change with overcontrol‐related measures would help ensure that future outcome measurement captures the processes that clients themselves experience as most important, alongside direct measurement of overcontrol. Future work might also examine the sensitivity to change and predictive validity of the FFOCI‐SF and related measures and compare the emerging factor structure across different overcontrol interventions to assess its broader clinical relevance.

## 5 Conclusion

This service review provides good psychometric support for the current outcome‐measurement framework used in GRO, while also highlighting areas for refinement. By demonstrating that most measures perform well and by identifying where further development is needed, the findings show how routine psychometric monitoring can strengthen both the quality and the ongoing evolution of the program. In addition, the findings contribute to a broader understanding of the construct of overcontrol itself, helping to clarify its key dimensions and their clinical relevance.

## Author Contributions

**Conal Twomey:** conceptualization, methodology, formal analysis, supervision, writing – original draft, writing – review and editing. **Amelia Nelson‐Sisinni:** conceptualization, data curation, investigation, project administration, writing – review and editing.

## Funding

The authors have nothing to report.

## Ethics Statement

As per hospital policy, ethical approval was not required as this is a service review. The service review was approved by St Patrick's Mental Health Services.

## Consent

Informed consent to use anonymised data for service review purposes was obtained from all participants at the point of entry into the GRO program.

## Conflicts of Interest

The authors declare no conflicts of interest.

## Supporting information


**Table S1:** Reliability (Cronbach’s Alpha; α) of FFOCI Subscales (pre‐intervention).

**Table S2:** Associations of FFOCI subscales with secondary measures addressing GRO’s core themes of overcontrol, and general distress.

**Table S3:** Exploratory Factor Analysis of the FFOCI‐SF Subscales: Three‐factor solution.

## Data Availability

Research data are not shared.

## References

[mpr70069-bib-0001] Adawi, M. , R. Zerbetto , T. S. Re , et al. 2019. “Psychometric Properties of the Brief Symptom Inventory in Nomophobic Subjects: Insights From Preliminary Confirmatory Factor, Exploratory Factor, and Clustering Analyses in a Sample of Healthy Italian Volunteers.” Psychology Research and Behavior Management 12: 145–154. 10.2147/prbm.S173282. 30881158 PMC6419603

[mpr70069-bib-0002] Alessandri, G. , M. Vecchione , B. M. Donnellan , N. Eisenberg , G. V. Caprara , and J. Cieciuch . 2014. “On the Cross‐Cultural Replicability of the Resilient, Undercontrolled, and Overcontrolled Personality Types.” Journal of Personality 82, no. 4: 340–353. 10.1111/jopy.12065. 23957593

[mpr70069-bib-0003] Block, J. H. , and J. Block . 1980. “The Role of Ego‐Control and Ego‐Resiliency in the Organization of Behavior.” In Development of Cognition, Affect and Social Relations: The Minnesota Symposia on Child Psychology, edited by W. A. Collins 13, 39–101. Erlbaum.

[mpr70069-bib-0004] Bohane, L. , N. Maguire , and T. Richardson . 2017. “Resilients, Overcontrollers and Undercontrollers: A Systematic Review of the Utility of a Personality Typology Method in Understanding Adult Mental Health Problems.” Clinical Psychology Review 57: 75–92. 10.1016/j.cpr.2017.07.005. 28850932

[mpr70069-bib-0005] Bonett, D. G. 2002. “Sample Size Requirements for Testing and Estimating Coefficient Alpha.” Journal of Educational and Behavioral Statistics 27, no. 4: 335–340. 10.3102/10769986027004335.

[mpr70069-bib-0006] Bonett, D. G. , and T. A. Wright . 2000. “Sample Size Requirements for Estimating Pearson, Kendall and Spearman Correlations.” Psychometrika 65, no. 1: 23–28. 10.1007/BF02294183.

[mpr70069-bib-0007] Booth, R. , and R. Egan . 2023. Group Radical Openness: An Intervention for Overcontrol. 1st ed. Routledge. 10.4324/9781003321576.

[mpr70069-bib-0008] Carter, N. T. , L. Guan , J. L. Maples , R. L. Williamson , and J. D. Miller . 2016. “The Downsides of Extreme Conscientiousness for Psychological Well‐Being: The Role of Obsessive Compulsive Tendencies.” Journal of Personality 84, no. 4: 510–522. 10.1111/jopy.12177. 25858019

[mpr70069-bib-0009] Clemente, M. J. , A. S. Martins Silva , M. O. Pozzolo Pedro , et al. 2022. “A Meta‐Analysis and Meta‐Regression Analysis of the Global Prevalence of Obsessive‐Compulsive Personality Disorder.” Heliyon 8, no. 7: e09912. 10.1016/j.heliyon.2022.e09912. 35865977 PMC9294057

[mpr70069-bib-0010] Collins, N. L. 1996. “Working Models of Attachment: Implications for Explanation, Emotion and Behavior.” Journal of Personality and Social Psychology 71, no. 4: 810–832. 10.1037/0022-3514.71.4.810. 8888604

[mpr70069-bib-0011] Crego, C. , D. B. Samuel , and T. A. Widiger . 2015. “The FFOCI and Other Measures and Models of OCPD.” Assessment 22, no. 2: 135–151. 10.1177/1073191114539382. 24963102

[mpr70069-bib-0012] Dana, D. 2018. The Polyvagal Theory in Therapy: Engaging the Rhythm of Regulation. W W Norton & Co.

[mpr70069-bib-0013] Derogatis, L. R. , and N. Melisaratos . 1983. “The Brief Symptom Inventory: An Introductory Report.” Psychological Medicine 13, no. 3: 595–605. 10.1017/s0033291700048017. 6622612

[mpr70069-bib-0014] Egan, R. , E. Long , J. McElvaney , and R. Booth . 2021. “Group Radical Openness: A Feasibility Study.” Counselling and Psychotherapy Research 22, no. 4: 913–924. 10.1002/capr.12480.

[mpr70069-bib-0015] Endermann, M. 2005. “The Brief Symptom Inventory (BSI) as a Screening Tool for Psychological Disorders in Patients With Epilepsy and Mild Intellectual Disabilities in Residential Care.” Epilepsy and Behavior 7, no. 1: 85–94. 10.1016/j.yebeh.2005.03.018. 15939672

[mpr70069-bib-0016] Gil, R. , J. Horcajo , P. Nájera , and M. A. Sorrel . 2024. “Navigating Ambiguity: Adapting and Validating the Personal Need for Structure Scale in Spanish.” Spanish Journal of Psychology 27: e22. 10.1017/sjp.2024.20. 39323223

[mpr70069-bib-0017] Gilmartin, T. , J. F. Dipnall , C. Gurvich , and G. Sharp . 2024. “Identifying Overcontrol and Undercontrol Personality Types Among Young People Using the Five Factor Model, and the Relationship With Disordered Eating Behaviour, Anxiety and Depression.” Journal of Eating Disorders 12, no. 1: 16. 10.1186/s40337-024-00967-4. 38267972 PMC10809654

[mpr70069-bib-0018] Gray, E. , N. Sweller , and S. Boag . 2024. “Child Abuse and Neglect and Obsessive–Compulsive Personality Traits: Effects of Attachment, Intolerance of Uncertainty, and Metacognition.” Journal of Child & Adolescent Trauma 17, no. 4: 1189–1209. 10.1007/s40653-024-00644-3. 39686931 PMC11646232

[mpr70069-bib-0019] Griffin, S. A. , T. Suzuki , D. R. Lynam , et al. 2018. “Development and Examination of the Five‐Factor Obsessive‐Compulsive Inventory‐Short Form.” Assessment 25, no. 1: 56–68. 10.1177/1073191116643818. 27095820

[mpr70069-bib-0020] Gross, J. J. , and O. P. John . 2003. “Individual Differences in Two Emotion Regulation Processes: Implications for Affect, Relationships, and Well‐Being.” Journal of Personality and Social Psychology 85, no. 2: 348–362. 10.1037/0022-3514.85.2.348. 12916575

[mpr70069-bib-0021] Hamtiaux, A. , and C. Houssemand . 2012. “Adaptability, Cognitive Flexibility, Personal Need for Structure, and Rigidity.” Journal of Psychology Research 2, no. 109: 563–585. 10.17265/2159-5542/2012.10.001.

[mpr70069-bib-0022] Hill, Jr. R. , S. C. South , and D. B. Samuel . 2025. “Measurement Invariance of the Five‐Factor Obsessive–Compulsive Inventory in a U.S. Census‐Matched Sample: Demographic Differences in Obsessive–Compulsive Personality Disorder Traits Across Age, Gender, and Education.” Personality Disorders: Theory, Research, and Treatment 17, no. 1: 83–91. 10.1037/per0000740. 40689914

[mpr70069-bib-0023] Isaksson, M. , A. Ghaderi , M. Wolf‐Arehult , and M. Ramklint . 2021. “Overcontrolled, Undercontrolled, and Resilient Personality Styles Among Patients With Eating Disorders.” Journal of Eating Disorders 9, no. 1: 47. 10.1186/s40337-021-00400-0. 33863394 PMC8052746

[mpr70069-bib-0024] Johnstone, V. , C. McDonough , R. Egan , K. Browne , and A. Corbett . 2024. “Group Radical Openness: Participants' Attributions of Change.” Counselling and Psychotherapy Research 24, no. 4: 1300–1309. 10.1002/capr.12750.

[mpr70069-bib-0025] Kłosowski, M. , J. Cieciuch , and W. Strus . 2019. “The Polish Adaptation of the Five‐Factor Obsessive‐Compulsive Inventory—Short Form (FFOCI‐SF): A Preliminary Study.” Health Psychology Report 7, no. 2: 165–175. 10.5114/hpr.2019.84214.

[mpr70069-bib-0026] Luo, J. , N. M. L. Wong , R. Zhang , et al. 2025. “A Network Analysis of Rumination on Loneliness and the Relationship With Depression.” Nature Mental Health 3, no. 1: 46–57. 10.1038/s44220-024-00350-x.

[mpr70069-bib-0027] Lynch, T. R. , R. J. Hempel , and C. Dunkley . 2015. “Radically Open‐Dialectical Behavior Therapy for Disorders of Over‐Control: Signaling Matters.” American Journal of Psychotherapy 69, no. 2: 141–162. 10.1176/appi.psychotherapy.2015.69.2.141. 26160620

[mpr70069-bib-0028] Lynch, T. R. , R. J. Hempel , B. Whalley , et al. 2020. “Refractory Depression ‐ Mechanisms and Efficacy of Radically Open Dialectical Behaviour Therapy (RefraMED): Findings of a Randomised Trial on Benefits and Harms.” British Journal of Psychiatry 216, no. 4: 204–212. 10.1192/bjp.2019.53. PMC7282863 31317843

[mpr70069-bib-0029] Mundfrom, D. J. , D. G. Shaw , and T. L. Ke . 2005. “Minimum Sample Size Recommendations for Conducting Factor Analyses.” International Journal of Testing 5, no. 2: 159–168. 10.1207/s15327574ijt0502_4.

[mpr70069-bib-0030] Neuberg, S. , and J. Newsom . 1993. “Personal Need for Structure: Individual Differences in the Desire for Simple Structure.” Journal of Personality and Social Psychology 65, no. 1: 113–131. 10.1037/0022-3514.65.1.113.

[mpr70069-bib-0031] Newman, D. A. 2014. “Missing Data: Five Practical Guidelines.” Organizational Research Methods 17, no. 4: 372–411. 10.1177/1094428114548590.

[mpr70069-bib-0032] Nolen‐Hoeksema, S. , B. E. Wisco , and S. Lyubomirsky . 2008. “Rethinking Rumination.” Perspectives on Psychological Science 3, no. 5: 400–424. 10.1111/j.1745-6924.2008.00088.x. 26158958

[mpr70069-bib-0033] Patrichi, A. , R. Rîmbu , A. C. Miu , and A. Szentágotai‐Tătar . 2025. “Loneliness and Emotion Regulation: A meta‐analytic Review.” Emotion 25, no. 3: 755–774. 10.1037/emo0001438. 39541515

[mpr70069-bib-0034] Porges, S. W. 2011. The Polyvagal Theory: Neurophysiological Foundations of Emotions, Attachment, Communication, and self‐regulation. W W Norton & Co.

[mpr70069-bib-0035] Preece, D. A. , R. Becerra , K. Robinson , and J. J. Gross . 2020. “The Emotion Regulation Questionnaire: Psychometric Properties in General Community Samples.” Journal of Personality Assessment 102, no. 3: 348–356. 10.1080/00223891.2018.1564319. 30714818

[mpr70069-bib-0036] Robins, R. W. , O. P. John , A. Caspi , T. E. Moffitt , and M. Stouthamer‐Loeber . 1996. “Resilient, Overcontrolled, and Undercontrolled Boys: Three Replicable Personality Types.” Journal of Personality and Social Psychology 70, no. 1: 157–171. 10.1037/0022-3514.70.1.157. 8558407

[mpr70069-bib-0037] Samuel, D. B. , A. D. Riddell , D. R. Lynam , J. D. Miller , and T. A. Widiger . 2012. “A five‐factor Measure of Obsessive‐Compulsive Personality Traits.” Journal of Personality Assessment 94, no. 5: 456–465. 10.1080/00223891.2012.677885. 22519829

[mpr70069-bib-0038] Schafer, J. L. , and J. W. Graham . 2002. “Missing Data: Our View of the State of the Art.” Psychological Methods 7, no. 2: 147–177. 10.1037/1082-989x.7.2.147. 12090408

[mpr70069-bib-0039] Shi, J. , L. Wang , and Y. Chen . 2009. “Validation of the Personal Need for Structure Scale in Chinese.” Psychological Reports 105, no. 1: 235–244. 10.2466/pr0.105.1.235-244. 19810450

[mpr70069-bib-0040] Tangney, J. P. , R. F. Baumeister , and A. L. Boone . 2004. “High Self‐Control Predicts Good Adjustment, Less Pathology, Better Grades, and Interpersonal Success.” Journal of Personality 72, no. 2: 271–324. 10.1111/j.0022-3506.2004.00263.x. 15016066

[mpr70069-bib-0041] Teixeira, R. C. R. , J. H. B. P. Ferreira , and A. B. C. Howat‐Rodrigues . 2019. “Collins and Read Revised Adult Attachment Scale (RAAS) Validity Evidences.” PSICO 50, no. 2: e29567. 10.15448/1980-8623.2019.2.29567.

[mpr70069-bib-0042] Tong, H. , W. K. Hou , L. Liang , T. W. Li , H. Liu , and T. M. C. Lee . 2021. “Age‐Related Differences of Rumination on the Loneliness‐Depression Relationship: Evidence From a Population‐Representative Cohort.” Innovation in Aging 5, no. 4: igab034. 10.1093/geroni/igab034. 34751252 PMC8522391

[mpr70069-bib-0043] Troisi, G. , A. Parola , and G. Margherita . 2022. “Italian Validation of AAS‐R: Assessing Psychometric Properties of Adult Attachment Scale‐Revised in the Italian Context.” Psychological Studies 67, no. 4: 605–613. 10.1007/s12646-022-00672-9. 36407970 PMC9663173

[mpr70069-bib-0044] Twomey, C. , S. Duffy , and R. Egan . 2025. The Comparative Effectiveness of a Group Therapy for Overcontrol (Group Radical Openness) when Delivered in‐person vs. Remotely. Submitted for Publication. 10.1002/jclp.70148 PMC13341044 42044294

[mpr70069-bib-0045] Volkert, J. , T. C. Gablonski , and S. Rabung . 2018. “Prevalence of Personality Disorders in the General Adult Population in Western Countries: Systematic Review and Meta‐Analysis.” British Journal of Psychiatry 213, no. 6: 709–715. 10.1192/bjp.2018.202. 30261937

