# Supplementary material for: Psychometric Evaluation of the FFOCI–SF and Other Clinical Outcome Measures in a Group Therapy for Overcontrol (Group Radical Openness)
Source: Int J Methods Psychiatr Res. 2026 Mar 7;35(1):e70069. doi: 10.1002/mpr.70069 (PMC12967023; doi:10.1002/mpr.70069)
Supplement: Supplementary file 1 — Table S1: Reliability (Cronbach’s Alpha; α) of FFOCI Subscales (pre‐intervention). [file MPR-35-e70069-s002.docx]

**Table 1.** Reliability (Cronbach’s Alpha; α) of FFOCI Subscales (pre-intervention).

| **Measure** | ***N*** | ***MV*** | **Mean (Maximum)** | ***SD*** | **(α)** |
| --- | --- | --- | --- | --- | --- |
| FFOCI-SF: Excessive Worry | 240 | 1 | 17.25 (20) | 2.85 | 0.77 |
| FFOCI-SF: Detached Coldness | 238 | 3 | 11.60 (20) | 3.59 | 0.75 |
| FFOCI-SF: Risk-Aversion | 234 | 7 | 14.67 (20) | 3.28 | 0.74 |
| FFOCI-SF: Constricted | 235 | 6 | 10.91 (20) | 3.63 | 0.67 |
| FFOCI-SF: Inflexible | 235 | 6 | 12.91 (20) | 3.33 | 0.69 |
| FFOCI-SF: Dogmatism | 227 | 14 | 12.47 (20) | 3.38 | 0.74 |
| FFOCI-SF: Perfectionism | 236 | 5 | 16.13 (20) | 3.22 | 0.77 |
| FFOCI-SF: Fastidiousness | 233 | 8 | 15.15 (20) | 3.51 | 0.79 |
| FFOCI-SF: Punctiliousness | 236 | 5 | 13.59 (20) | 3.67 | 0.78 |
| FFOCI-SF: Workaholism | 236 | 5 | 12.28 (20) | 3.57 | 0.73 |
| FFOCI-SF: Doggedness | 237 | 4 | 13.06 (20) | 4.07 | 0.81 |
| FFOCI-SF: Ruminative Deliberation | 238 | 3 | 15.36 (20) | 3.46 | 0.84 |
| **Note.** FFOCI-SF = Five Factor Obsessive Compulsive Inventory – Short Form. | | | | | |
