# Supplementary material for: Psychometric Evaluation of the FFOCI–SF and Other Clinical Outcome Measures in a Group Therapy for Overcontrol (Group Radical Openness)
Source: Int J Methods Psychiatr Res. 2026 Mar 7;35(1):e70069. doi: 10.1002/mpr.70069 (PMC12967023; doi:10.1002/mpr.70069)
Supplement: Supplementary file 2 — Table S2: Associations of FFOCI subscales with secondary measures addressing GRO’s core themes of overcontrol, and general distress. [file MPR-35-e70069-s003.docx]

**Table 2.** Associations of FFOCI subscales with secondary measures addressing GRO’s core themes of overcontrol, and general distress.

| **FFOCI subscale** | **Theme: Distance in Relationships** | | | **Theme: Rigidity** | | **Theme: Emotional Inhibition** | | **General Distress** |
| --- | --- | --- | --- | --- | --- | --- | --- | --- |
|  | **RAAS Close** | **RAAS Depend** | **RAAS Anxiety** | **PNS Desire for structure** | **PNS Response to lack of structure** | **ERQ Cognitive reappraisal** | **ERQ Expressive Suppression** | **BSI Total** |
| FFOCI-SF: Excessive Worry | -0.21** | -0.23** | 0.36** | 0.18** | 0.43** | -0.14* | 0.06 | 0.50** |
| FFOCI-SF: Detached Coldness | -0.45** | -0.13* | 0.12 | 0.15* | 0.24** | -0.10 | 0.27** | 0.13 |
| FFOCI-SF: Risk-Aversion | -0.19** | 0.04 | 0.09 | 0.47** | 0.57** | 0.03 | 0.06 | 0.09 |
| FFOCI-SF: Constricted | -0.22** | 0.03 | -0.02 | 0.04 | 0.02 | -0.05 | 0.25** | -0.05 |
| FFOCI-SF: Inflexible | -0.21** | 0.02 | 0.13 | 0.44** | 0.54** | 0.01 | 0.12 | 0.07 |
| FFOCI-SF: Dogmatism | -0.22** | -0.20** | 0.24** | 0.38** | 0.45** | 0.07 | 0.16* | 0.28** |
| FFOCI-SF: Perfectionism | -0.09 | -0.11 | 0.15* | 0.43** | 0.35** | 0.02 | 0.06 | 0.16* |
| FFOCI-SF: Fastidiousness | -0.15* | -0.15* | 0.20** | 0.45** | 0.37** | 0.01 | 0.07 | 0.19** |
| FFOCI-SF: Punctiliousness | -0.22** | -0.12 | 0.21** | 0.45** | 0.44** | -0.01 | 0.12 | 0.14 |
| FFOCI-SF: Workaholism | -0.06 | -0.11 | 0.06 | 0.23** | 0.18** | 0.07 | 0.13 | 0.06 |
| FFOCI-SF: Doggedness | -0.13 | -0.07 | 0.09 | 0.38** | 0.32** | 0.03 | 0.12 | 0.16* |
| FFOCI-SF: Ruminative Deliberation | -0.23** | -0.14* | 0.26** | 0.35** | 0.45** | 0.05 | 0.13* | 0.33** |

**Note.** *Correlation is significant at the 0.05 level (2-tailed); **Correlation is significant at the 0.01 level (2-tailed). BSI = Brief Symptom Inventory; ERQ = Emotional Regulation Questionnaire; FFOCI-SF = Five Factor Obsessive Compulsive Inventory – Short Form; PNS = Personal Need for Structure; RAAS = Revised Adult Attachment Scale.
