# Supplementary material for: Psychometric Evaluation of the FFOCI–SF and Other Clinical Outcome Measures in a Group Therapy for Overcontrol (Group Radical Openness)
Source: Int J Methods Psychiatr Res. 2026 Mar 7;35(1):e70069. doi: 10.1002/mpr.70069 (PMC12967023; doi:10.1002/mpr.70069)
Supplement: Supplementary file 3 — Table S3: Exploratory Factor Analysis of the FFOCI‐SF Subscales: Three‐factor solution. [file MPR-35-e70069-s001.docx]

**Table 3.** Exploratory Factor Analysis of the FFOCI-SF Subscales: Three-factor solution.

| **F1: Excessively High Standards (α = 0.88)** | | | **F2: Intolerance of Uncertainty (α = 0.75)** | | | **F3: Disconnection from Self and Others (α = 0.76)** | | |
| --- | --- | --- | --- | --- | --- | --- | --- | --- |
| **Code** | **Subscale** | **Loading** | **Code** | **Subscale** | **Loading** | **Code** | **Subscale** | **Loading** |
| C4 | Workaholism. | 0.93 | C6 | Ruminative Deliberation. | 0.74 | O3 | Constricted. | 0.78 |
| O6 | Doggedness. | 0.91 | N1 | Excess Worry. | 0.68 | E1 | Detached Coldness. | 0.68 |
| C1 | Perfectionism. | 0.73 | E5 | Risk Aversion. | 0.62 |  |  |  |
| C2 | Fastidiousness. | 0.65 | O4 | Inflexible. | 0.45 |  |  |  |
| C3 | Punctiliousness. | 0.45 |  |  |  |  |  |  |
| C5 | Dogmatism. | 0.38 |  |  |  |  |  |  |

**Note.** F = Factor.
